# Extracorporeal photopheresis as a therapeutic approach for treatment resistant immune-related adverse events in anti-PD-1-treated melanoma patients

**DOI:** 10.3389/fimmu.2025.1727312

**Published:** 2025-12-16

**Authors:** Carl Maximilian Thielmann, Johanna Andrea Seier, Lisa Schielke, Lea Jessica Albrecht, Lisa Zimmer, Elisabeth Livingstone, Anne Zaremba, Georg Lodde, Joachim Dissemond, Wiebke Sondermann, Frederik Krefting, Alpaslan Tasdogan, Alexander Roesch, Eva Hadaschik, Florian Rambow, Klaus Griewank, Selma Ugurel, Dirk Schadendorf, Jan-Malte Placke

**Affiliations:** 1 Department of Dermatology, Venereology and Allergology, University Hospital Essen, University of Duisburg-Essen, Germany & German Cancer Consortium (DKTK), Partner Site, Essen, Germany; 2 Department of Applied Computational Cancer Research, IKIM, University Hospital Essen, Essen, Germany; 3 Department of Dermatology, Bielefeld University, Medical School and University Medical Center OWL, Klinikum Bielefeld Rosenhöhe, Bielefeld, Germany; 4 Research Alliance Ruhr, Research Center One Health, University Duisburg-Essen, Essen, Germany; 5 National Center for Tumor Diseases (NCT-West), Essen, Germany

**Keywords:** extracorporeal photopheresis, immune checkpoint inhibitors, immune related colitis, immune related hepatitis, melanoma

## Abstract

**Background:**

Checkpoint inhibition induced immune-related adverse events (irAE) may be steroid-dependent or steroid-refractory and are associated with increased morbidity, mortality and potentially compromised anti-tumor immunity. Extracorporeal photopheresis (ECP) has emerged as an alternative for salvage therapy, however, evidence remains scarce.

**Methods:**

This monocenter retrospective study included patients with either irColitis or irHepatitis, who received ECP after failure or dependence on high-dose corticosteroids + infliximab/vedolizumab or mycophenolate mofetil/tacrolimus. Clinical activity was quantified at least weekly (stool frequency for colitis; AST/ALT for hepatitis) and primary endpoint was change in irAE activity over time. Secondary analyses included steroid-sparing, overall safety, and melanoma-specific outcomes. Spearman’s correlation assessed irAE severity reduction.

**Results:**

Six patients were included in this study (irColitis n = 4; irHepatitis n = 2; CTCAE ≥ 3). Extracorporeal photopheresis was started after initial therapy with corticosteroids and immunosuppression was not successful. All ECP cycles included two consecutive treatment days. irAE activity declined promptly after ECP across patients: irColitis showed strong negative correlation with time since ECP (r_s_ range -0.88 to -0.97); irHepatitis displayed parallel ALT/AST declines (r_s_ ≥ -0.92). Corticosteroids were tapered following ECP start with a median corticosteroid reduction across all patients to 25% of baseline dose (IQR: 20.7 - 33.3) by week 4 and to <5% of baseline dose by week 9 (IQR: 1.6 - 4.7). No ECP-related adverse events were observed. Accelerated disease progression was not observed during or after ECP.

**Conclusions and relevance:**

This study of six patients with irColitis or irHepatitis provides evidence that use of ECP is associated with clinical remission and steroid sparing, while demonstrating an excellent safety profile and not compromising disease control. Our data supports the use of ECP as salvage therapy for steroid- and immunosuppression- refractory irAE in cancer patients.

## Introduction

Advanced melanoma has undergone a therapeutic revolution over the course of the past two decades, driven by immune checkpoint inhibitors (ICI), targeting cytotoxic T-lymphocyte-associated protein 4 (CTLA-4) and programmed cell death-1 (PD-1), as well as BRAF-/MEK- targeted therapies. Combination of anti-PD-1 and anti-CTLA-4 therapies have achieved 50% overall survival (OS) for patients with advanced metastatic disease in clinical trials establishing ICI as standard therapy in advanced or metastatic melanoma (1).

Although clinically beneficial, the enhanced immune activation that causes the potent anti-tumor immunity has a downside: ICI may induce a broad spectrum of immune-related adverse events (irAE) (2). Side effects can affect any organ system, with skin, gastrointestinal tract, lungs, liver, and endocrine glands being most commonly involved (3). Patients receiving anti-PD-1 (or anti-PD-L1) antibody monotherapy have a lower incidence of any-grade irAE compared to those patients treated with combination therapies (e.g., ipilimumab + nivolumab). Additionally, the type of irAE differs depending on the type of ICI (3–5). irAE are graded according to the definition of the Common Terminology Criteria for Adverse Events (CTCAE).

Managing ICI-induced toxicities can be challenging, as irAEs can cause treatment interruptions and increases in morbidity and mortality. Patients present with diverse clinical symptoms, which demand ongoing awareness and prompt intervention. The central element in the treatment of ICI-induced toxicity is immunosuppressive therapy, with corticosteroids being considered first-line therapy for moderate to severe irAE (6). American and European guidelines recommend prompt initiation of corticosteroids (i.e., prednisone 1-2mg/kg) for CTCAE grade ≥3 toxicity in combination with discontinuation of therapy to prevent aggravation of immune-mediated tissue damage (6, 7). Although many patients show sufficient responses to corticosteroid therapies, a subset of patients experience immune toxicities which are steroid-refractory (failing to improve on corticosteroid therapy) or steroid-dependent (initially responding, but flaring as corticosteroids are tapered) (8–10). In gastrointestinal toxicity particularly, a significant fraction of irColitis cases (30-40% of cases) do not resolve with corticosteroids alone (11). These steroid-refractory cases pose a clinical challenge, as prolonged courses of high-dose corticosteroids carry substantial risk of increased morbidity, including opportunistic infections, metabolic complications and preclude patients from resuming their therapeutic regimen leading to potential compromise on cancer control (12–15). Yet, there is no consensus on optimal management once both corticosteroids and standard second-line agents (e.g., infliximab, vedolizumab, mycophenolate mofetil [MMF]) fail to achieve remission.

Extracorporeal photopheresis (ECP) has emerged as a potential salvage immunomodulatory therapy for patients, whose severe irAE present treatment challenges. ECP, which was originally developed for cutaneous T-cell lymphoma, is an apheresis-based photochemotherapy, in which leukocytes are isolated from the blood, treated with photosensitizer 8-methoxypsoralen, subsequently exposed to UV-A light for induction of apoptosis and reinfused to the patient (16). While its precise mechanism remains not fully understood to date, emerging data shows that ECP may mitigate irAE via an adiponectin-mediated, M2-phenotypic macrophage response: ECP-treated apoptotic leukocytes undergo phagocytosis by macrophages, polarizing these phagocytes to an M2-like anti-inflammatory phenotype, activating anti-inflammatory mediators (e.g., adiponectin, CD206). M2-polarization and adiponectin induction collectively mitigate inflammation by reducing pro-inflammatory cytokines, while preserving anti-tumor immunity through suppression of Th1/Tc1 and tissue-resident memory T-cell populations (17).

Over the past decades, ECPs applications have expanded to a variety of T-cell mediated disorders, e.g., for steroid-refractory graft-versus-host disease (GvHD) after allogeneic stem cell transplantation. Generally, ECP is considered to have an excellent safety profile (18–20). Unlike corticosteroids, ECP is not associated with significant infection risk or end organ toxicity. This favorable risk-benefit profile has prompted exploration of ECP as a rescue therapy for severe irAEs.

To date, initial clinical data on the use of ECP in ICI-induced toxicities remained limited and consisted mainly of case reports and case series (21–23). Previous treatment successes, coupled with the immunologic advantages of ECP, have paved the way for prospective trials: a first clinical trial is underway to compare ECP against other second-line immunosuppressants for steroid-refractory irAEs, with the results from interim analysis only recently published (19). Given the clinical imperative to find safe and effective therapies for steroid-refractory or -dependent irAEs, further data on ECP usage for irAE is warranted. To contribute to closing that gap, we here report a single-center retrospective study of six patients with metastatic melanoma who developed severe immune-related colitis (irColitis) or hepatitis (irHepatitis) during anti-PD-1 based ICI. Each patient was treated with adjunct ECP after failing to respond to high-dose corticosteroids and additional immunosuppressants. We here describe their clinical courses and outcomes and highlight the role of ECP in achieving symptom control. This series aims to add to the limited literature on ECP as a salvage therapy for irAEs and its consideration as an immunomodulatory option in managing life-threatening, steroid-refractory toxicity in melanoma patients.

## Materials and methods

### Patient identification

We screened melanoma patients (with advanced or metastatic disease) who received anti-PD-1 based ICI and experienced at least one severe (grade ≥3; graded according to CTCAE version 6.0), steroid-unresponsive (as per SITC consensus definition (10)) irAE (either colitis or hepatitis), for which patients were treated with ECP between 03/2020 and 07/2025. Patients with available clinical follow-up data were included in this study (n = 6) with one patient being excluded for being included in a recently published multicenter trial by Braun et al. (17). Patient characteristics were obtained from electronic medical records from Essen University Hospital. All patients gave written informed consent prior to this analysis. The study was conducted in accordance with the Declaration of Helsinki and was approved by the local ethics committee of the University of Duisburg-Essen (23-11502-BO).

### Patient involvement

It was not possible to involve patients or the public in the design, conduct, or reporting of our research, given its retrospective nature.

### Procedural details of extracorporeal photopheresis

After informed written consent, ECP was performed using the Therakos™ CellEx™ system following the manufacturer protocol. ECP sessions were conducted on two consecutive days, with treatment frequency adjusted based on clinical response and urgency. For the procedure, blood was drawn from the patients via peripheral venous access. Separation of leukocytes and erythrocytes was achieved through centrifugation. Following separation, leukocytes were treated with 8-methoxypsoralen (8-MOP) at a concentration of 200 ng/mL and subsequently irradiated with UV-A light at 2 J/cm^2^. The irradiated leukocytes were then reinfused to the patient. Each treatment session lasted approximately 2-3 hours.

### Treatment protocols

Patients received ICI according to standard institutional protocols. Combination therapy consisted of ipilimumab 3 mg/kg plus nivolumab 1 mg/kg intravenously every 3 weeks for 4 cycles, followed by nivolumab maintenance at 480 mg (flat dose) intravenously every 4 weeks. Nivolumab monotherapy was administered at 480 mg (flat dose) intravenously every 4 weeks. Pembrolizumab monotherapy was administered at 200 mg (flat dose) intravenously every 3 weeks. For management of steroid-refractory irAE, infliximab was administered at 5 mg/kg body weight intravenously. If not otherwise stated in patient vignettes, standard dosing was applied.

### Study outcomes

The primary outcome was change in irAE activity following ECP initiation, quantified as daily stool frequency for irColitis and serum transaminase levels (ALT/AST) for irHepatitis. irAE intensity was assessed longitudinally from irAE onset through follow-up.

### Statistical analysis

Associations between tumor origin and clinical parameters were examined using Chi-square tests or Fisher’s exact tests, as appropriate and indicated. Continuous variables are reported as means with standard deviations or as medians with interquartile ranges, as applicable, while categorical variables are expressed as counts and percentages. Statistical analyses of correlation were performed using Spearman’s correlation. Statistical analyses were conducted using Microsoft Excel (Redmond, Washington, USA), R (version 4.4.2), RStudio (2024.12.1). A p-value < 0.05 was considered statistically significant.

## Results

### Cohort overview

**Figure 1:**
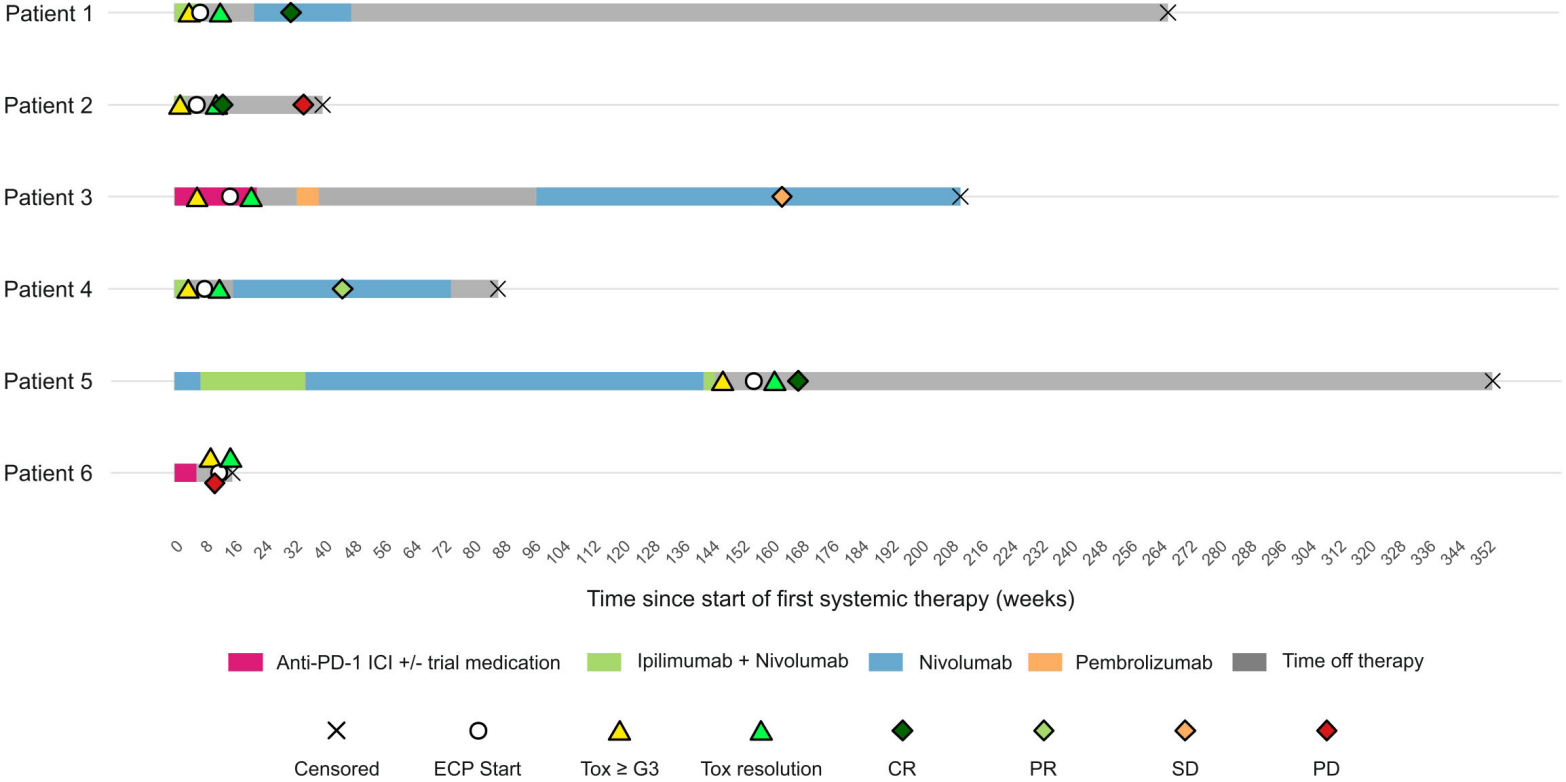
Swimmer plot showing individual patient characteristics (n = 6) with respect to time since start of first systemic therapy including: treatment type and duration, overall survival (OS), censorship date, and timing of responses of either complete response (CR), partial response (PR), stable disease (SD) or progressive disease (PD). Response evaluation time points indicate best overall response (BOR) achieved during ICI therapy. Timing of toxicities of grade 3 or above, ECP start, and resolution of toxicity (CTCAE grade ≤1) are also noted.

Six melanoma patients with irAE (CTCAE grade ≥ 3; hepatitis n = 2; colitis n = 4; **Tables 1**, **2**) were identified, who received ECP after inadequate response to high-dose corticosteroids and additional second-line immunosuppressants (+ infliximab/vedolizumab or MMF/tacrolimus). ECP began a median ~4 weeks after initial irAE onset (range ~1-10). All cycles followed the standard manufacturer protocol with two consecutive treatment days, applying 4 (± 1.2 SD) ECP cycles until resolution of symptoms (irAE severity CTCAE grade ≤ 1). No ECP-related adverse events occurred. Overview of treatment courses, clinical responses, onset of irAE and ECP start is shown in **Figure 1**.

**Table 1:** Clinical characteristics of ECP treated patients (n = 6).

Variable, n (%)	
Age, n (%)	
Median	56
Range	47 - 66
≤60 years	4 (66.7)
>60 years	2 (33.3)
Sex, n (%)	
Female	1 (16.7)
Male	5 (83.3)
Primary AJCC (2017) stage at first diagnosis, n (%)	
II	2 (33.3)
IIIB/C	1 (16.7)
IV	3 (50.0)
Treatment setting, n (%)	
Metastatic	6 (100.0)
Therapy regimen, n (%)	
Ipilimumab + nivolumab	4 (66.7)
Clinical trial (anti-PD-1 based)	2 (33.3)
ICI treatment, n (%)	
Stopped	3 (50.0)
Rechallenged	3 (50.0)
Second-line therapy for irAE, n (%)	
ECP	6 (100.0)
Infliximab	3 (50.0)
MMF + tacrolimus	2 (33.3)
Vedolizumab	1 (16.7)
irAE type, n (%)	
Hepatitis	2 (33.3)
Colitis	4 (66.7)
irAE CTCAE grade – before treatment, n (%)	
1-2	0 (0.0)
3-4	6 (100.0)
irAE CTCAE grade – after treatment, n (%)	
1-2	6 (100.0)
3-4	0 (0.0)
Additional irAE, n (%)	
Maculopapular rash	3 (50.0)
Pruritus	1 (16.7)
Flu-like symptoms	2 (33.3)
Heart failure	1 (16.7)

**Table 2:** Detailed clinical characteristics of ECP treated patients (n = 6).

Patient	1	2	3	4	5	6
Stage (AJCC, eighth edition)	IV	IV	IV	IV	IV	IIIC
Age	47	66	64	55	47	56
Sex	Male	Male	Male	Male	Male	Female
irAE	Colitis	Colitis	Colitis	Colitis	Hepatitis	Hepatitis
Other irAE	Maculopapular rash, pruritus	heart failure, maculopapular rash, and flu-like symptoms	N/A	Maculopapular rash, flu-like Symptoms	N/A	N/A
ICI regimen	Ipilimumab/Nivolumab	Ipilimumab/Nivolumab	Anti-PD1/Anti-CTLA4	Ipilimumab/Nivolumab	Ipilimumab/Nivolumab	Anti-PD1
Immunosuppressants used	CS, Infliximab	CS, Infliximab, Vedolizumab	CS, Infliximab	CS, Infliximab	CS, MMF, Tacrolimus	CS, MMF
Previous ICI therapy	N/A	N/A	N/A	N/A	Nivolumab mono, Ipilimumab/Nivolumab	N/A
ICI re-challenge	Yes	No	Yes	Yes	No	No

### Case-by-case view

#### Patient 1

A 47-year-old male with metastatic melanoma of the right ear helix (stage IV since January 2020 with lung, subcutaneous, and bone metastases) received cycles of ipilimumab plus nivolumab every three weeks. After two cycles in March 2020, he developed grade 4 irColitis (≥30 bloody/watery stools/day). Besides irColitis, he developed a maculopapular rash (G1) and pruritus (G1). Methylprednisolone 80 mg/day (1 mg/kg) was started, which was later escalated to 160 mg/day (2 mg/kg) after 48 hours without apparent clinical improvement. As the symptoms persisted, two doses of infliximab were given in a 10-day interval without meaningful improvement, which led to the decision to start ECP and oral budesonide.

ECP initiation was four weeks after colitis onset, while he remained on 80 mg methylprednisolone/day and 9 mg oral budesonide. ECP was administered weekly for the first three cycles, with a two-week interval before the fourth cycle. Stool frequency decreased to ~15/day after the first week and to seven/day by completion of the second week after ECP start. Complete resolution occurred before initiation of the fourth cycle (weekly to bi-weekly regimen). During ECP, corticosteroids were tapered subsequently with reduction to 20 mg/day after four cycles. No ECP-related adverse events were recorded.

After the patient did not experience any recurrence of diarrhea, nivolumab maintenance was started in July 2020 (three doses given). Immune related diarrhea recurred in November 2020 and January 2021. Each flare was controllable with additional single cycles of ECP (December-July 2021) while nivolumab was paused. A PET-CT in September 2020 showed complete metabolic response. At last follow-up in August 2025, the patient remains disease-free and asymptomatic off all systemic immunosuppression.

#### Patient 2

A 66-year-old male was diagnosed with stage IV melanoma in 2024 (metastases in lymph nodes and lung) and received a single dose of ipilimumab and nivolumab in October 2024. Within ten days he developed severe diarrhea, with >20 watery, partially bloody stools/day (CTCAE grade 4). Besides irColitis, he developed heart failure (G3), maculopapular rash (G2), and flu-like symptoms (G1). Since the patient was abroad, oral instead of iv methylprednisolone 80 mg/day was started for four days and escalated upon hospital admission to 240 mg/day methylprednisolone (2 mg/kg). Additionally, the patient received infliximab. This combination therapy initially led to an improvement of diarrhea. However, symptoms rebounded whenever the corticosteroid dose was reduced to doses below 120 mg. He ultimately received three doses weekly infliximab without sustained benefit.

ECP was initiated four weeks after colitis onset. At that point he was passing ~15 stools/day while still receiving daily methylprednisolone (1 mg/kg). ECP was delivered weekly in five consecutive cycles (weekly regimen). Adjunctive vedolizumab (two doses of 300 mg; one week and three weeks after ECP start) and oral budesonide (3 mg, four times daily) were added. Clinical response paralleled ECP initiation: stool frequency fell to six/day within a week, while a lasting reduction was observed after four ECP cycles. No ECP-related adverse events occurred. Seven weeks post-ECP, the patient noticed recurring diarrhea, for which he received a single dose vedolizumab on the following day, leading to symptom resolution shortly afterwards. The patient has been without recurrence since.

Anti-PD-1 based ICI was withheld after the single induction dose. Interim PET-CT (May 2025) revealed a subcutaneous nodule on the abdomen, which was excised and consistent with subcutaneous metastasis. Since then, the patient has remained disease-free and asymptomatic after starting ECP.

#### Patient 3

A 64-year-old male with stage IV acral-lentiginous melanoma (distant lymph node metastases) entered a clinical trial and received anti-PD1/-CTLA4 based study medication every four weeks, following the clinical trial protocol. After two cycles he developed irColitis (CTCAE grade 3, 5-10 watery stools/day), confirmed by biopsy, that required repeated hospital admissions despite daily methylprednisolone doses of up to 2mg/kg. Because symptoms persisted over >8 weeks of corticosteroids and two doses of weekly infliximab, ECP was initiated, and four cycles were delivered every two weeks over the course of six weeks. At ECP start he remained on methylprednisolone, which was discontinued shortly after the fourth ECP cycle. Colitis briefly flared to eight stools/day after the first cycle but fell to three stools by the second event and ≤2 stools thereafter, without further escalation of therapy. No ECP-related adverse events occurred.

ICI was paused during ECP. Afterwards, the patient was re-exposed to anti-PD-1 based monotherapy with pembrolizumab and, subsequently, nivolumab. Latest staging in early 2025 showed stable disease. At last follow-up in July 2025, the patient remains off systemic corticosteroids and has not experienced further gastrointestinal irAEs.

#### Patient 4

A 55-year-old male with stage IV melanoma of the back with distant metastasis (an 8 cm mass in the left abdomen in the wall of a small intestine loop) as well as lymph node metastases (mesenteric, inguinal, and iliac) began combination therapy with ipilimumab and nivolumab in October 2023. After two doses he developed CTCAE grade 3-4 irColitis. Besides, he developed a maculopapular rash (G1) and flu-like symptoms (G1). High-dose intravenous methylprednisolone 80 mg/day (1 mg/kg) was started upon hospital admission. Infliximab and oral budesonide (3 mg, three times daily) were added after 5 days, but diarrhea persisted. ECP was started after four weeks without clinical improvement and was delivered weekly for four cycles. Frequency of watery stools fell to one/day before the second cycle and to zero thereafter. This allowed a stepwise corticosteroid taper for 6 weeks followed by complete discontinuation. No ECP-related adverse events were observed, and no additional immunosuppressants were required.

The patient received maintenance therapy with nivolumab after cessation of symptoms. Anti-PD1 based ICI was paused early 2025; one year after surveillance imaging has shown a complete metabolic response. At last follow-up in August 2025 (20 months post-ECP) the patient remains disease-free, off all immunotherapy and immunosuppression, with sustained remission of colitis.

#### Patient 5

A 47-year-old male with metastatic scrotal melanoma (epicardial metastasis diagnosed in July 2018) initially received nivolumab monotherapy. After disease progression he was switched to combination therapy of ipilimumab and nivolumab. After three cycles (standard dose) the patient developed irHepatitis, which responded to a four-month taper of methylprednisolone. Maintenance nivolumab was resumed, but new brain metastases in May 2021 led to re-exposition to ipilimumab and nivolumab with two further cycles. Six weeks after re-exposition he was admitted with irHepatitis (CTCAE grade 3, confirmed by biopsy).

High-dose methylprednisolone (up to 2 mg/kg) plus MMF and later, tacrolimus was not sufficient to achieve clinically meaningful control of hepatitis, as transaminase levels remained markedly elevated, leading to the decision to start ECP. ECP was started seven weeks after hepatitis onset in a biweekly regimen (for two cycles, remaining cycles with four-week intervals), while he remained on triple immunosuppression (corticosteroids/MMF/tacrolimus). Four ECP cycles were administered in total. ALT and AST steadily declined and were within normal limits after three cycles. Systemic immunosuppression was tapered in parallel: corticosteroids/MMF/tacrolimus doses were subsequently reduced; methylprednisolone was stopped shortly after the fourth ECP cycle. No ECP-related adverse events occurred.

Anti-PD-1 based ICI was permanently discontinued. A PET-CT in December 2021 demonstrated complete metabolic response, which has been sustained on surveillance imaging through July 2025 (45 months post-ECP) without further immunotherapy or immunosuppression. Liver enzymes remain within normal limits and no recurrent irAEs were observed.

#### Patient 6

A 56-year-old female with stage IIIC melanoma entered a clinical trial in March 2025 and received four doses of anti-PD1-based drug regimen every three weeks. Early June she was admitted with CTCAE grade 3 irHepatitis, confirmed via hepatic biopsy. High-dose methylprednisolone of 160 mg/day (2 mg/kg) was started upon admission, MMF (1g, two times a day) was added seven days later. MMF allowed methylprednisolone to be tapered to 1 mg/kg. CT imaging showed new cervical and left axillary metastases (leading to trial discontinuation) and raised strong suspicion of a sigmoid colon carcinoma for which surgery was consulted (surgery advised and feasible under corticosteroids and ECP but not under MMF). To achieve normalization of transaminases and allow for MMF wean, weekly ECP (two consecutive treatment days per cycle) was started end of June 2025. After four cycles MMF was stopped, with subsequent reduction of methylprednisolone to 20 mg/day. Transaminases improved in parallel with improvement after four cycles. No ECP-related adverse events occurred.

Given metastatic progression, anti-PD1 based ICI was discontinued with a planned transition to BRAF/MEK inhibitors. The patient remains on weekly ECP with continued corticosteroid taper while preparing for sigmoid resection as recommended by the interdisciplinary tumor board.

### Symptom decline and corticosteroid tapering

**Figure 2:**
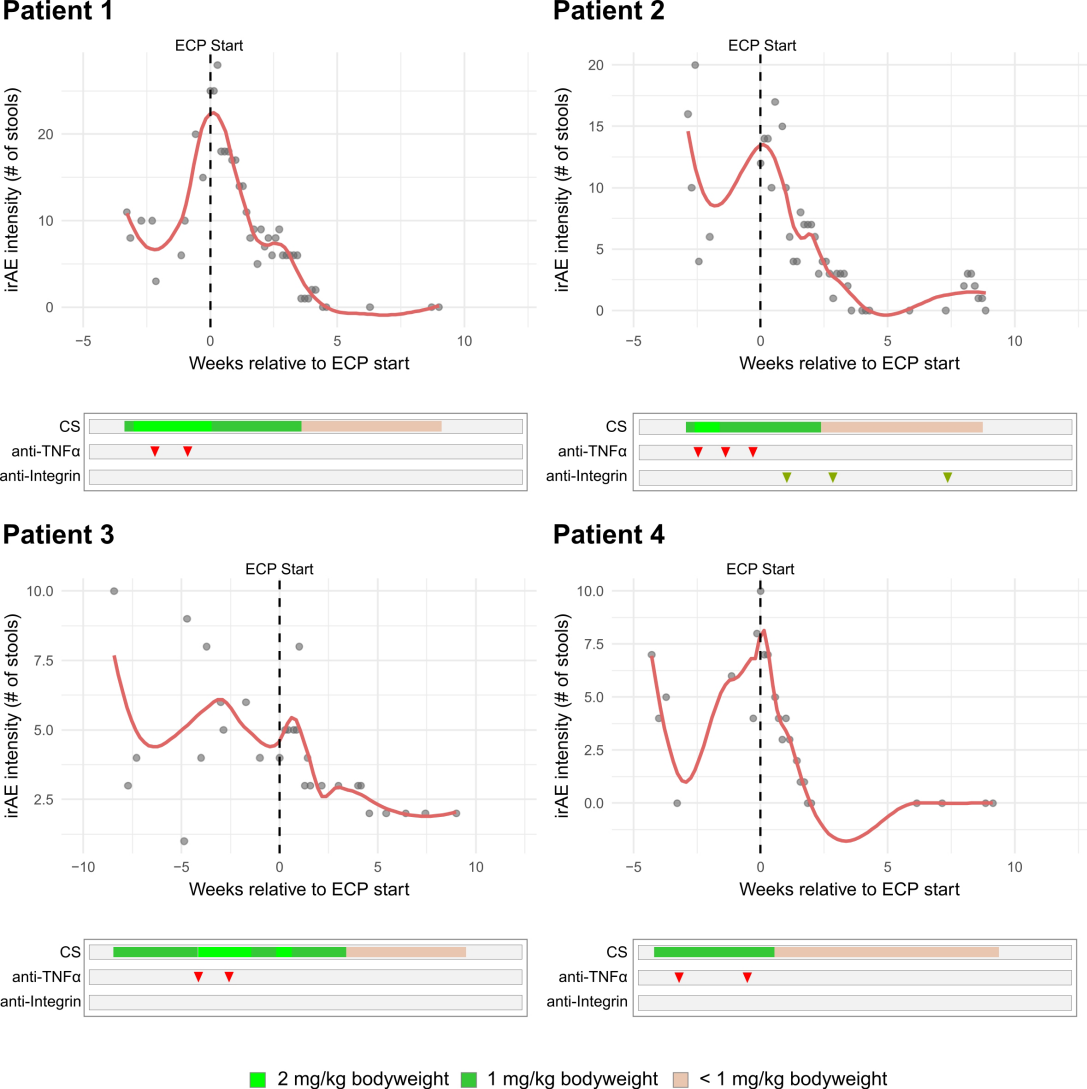
Longitudinal plots for included patients (n = 4) with irColitis. irAE severity was quantified by daily stool frequency. The course of corticosteroid (CS), anti-TNFα, and anti-Integrin treatments are shown.

**Figure 3:**
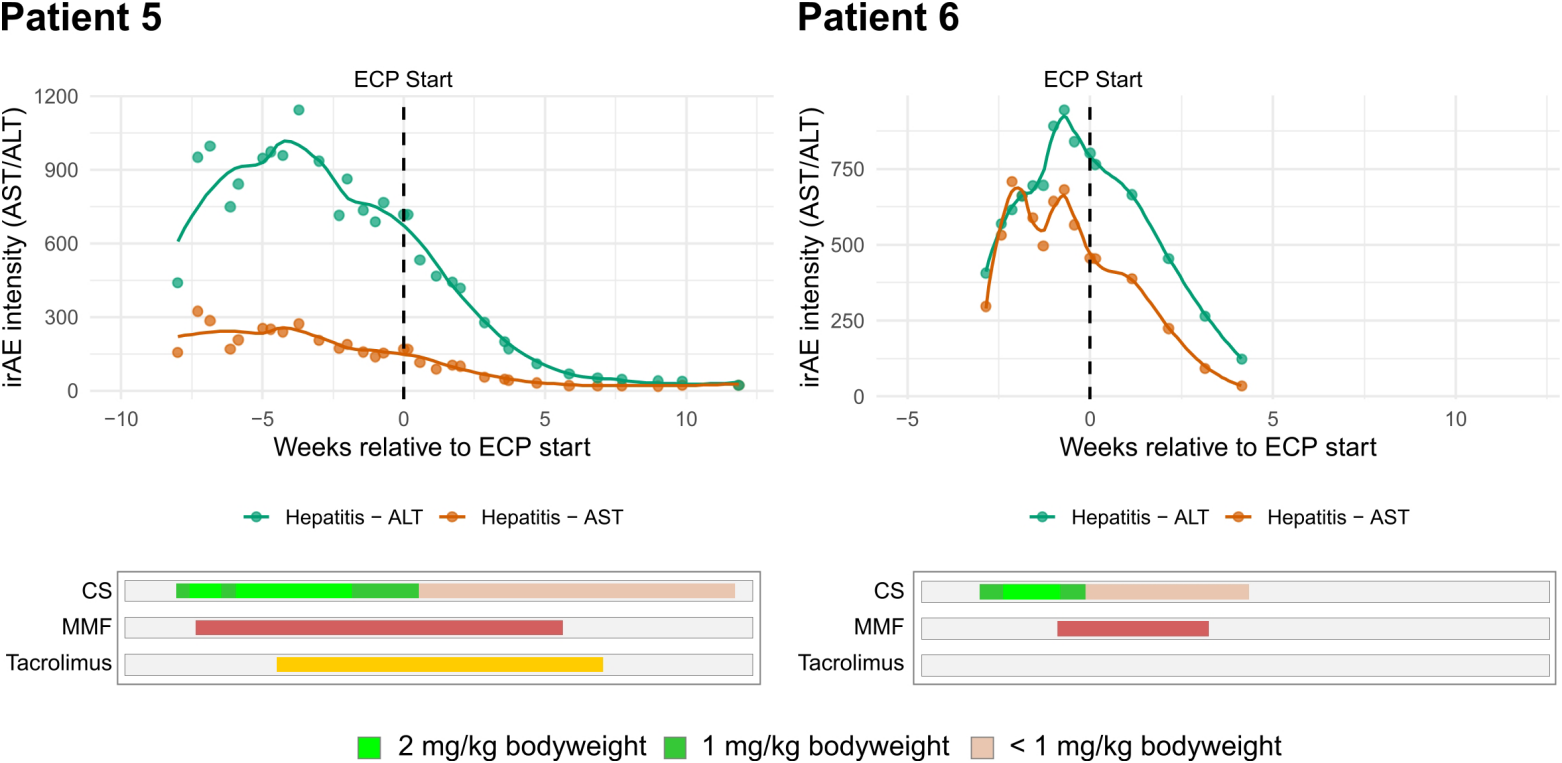
Longitudinal plots for included patients (n = 2) with irHepatitis. irAE severity was assessed by alanine aminotransferase (ALT) and aspartate aminotransferase (AST) levels. The course of corticosteroid (CS), mycophenolate mofetil (MMF), and tacrolimus treatments are shown.

**Figure 4:**
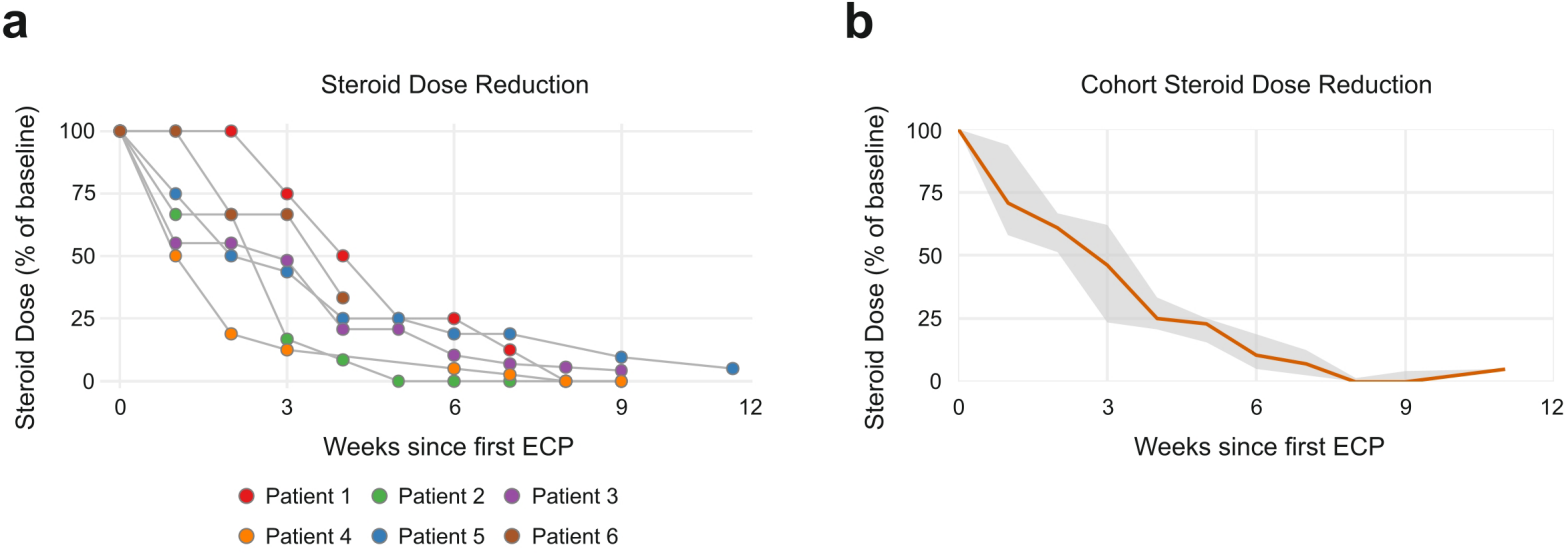
**(a)** Patient-level trajectories of corticosteroid tapering following initiation of ECP. **(b)** Cohort-level trend of systemic corticosteroid dose over time, expressed as percentage of baseline dosage.

Across all patients, irAE activity declined consistently after starting ECP (**Figures 2**, **3**). In the four cases presenting with irColitis, weekly intensity scores (as # of stools/day) showed strong negative correlation with time (patient 1: r_s_ = -0.96; p = 1.1 x 10^-19^; patient 2: r_s_ = -0.88; p = 1.31 x 10^-12^; patient 3: r_s_ = -0.90; p = 4.26 x 10^-7^; patient 4: r_s_ = -0.97; p = 1.05 x 10^-10^). In the two cases presenting with irHepatitis, transaminases decreased almost in parallel across ALT/AST (patient 5 – ALT: r_s_ = -1.00, p = 3.1 x 10^-21^, AST: r_s_ = -0.92, p = 6.3 x 10^-7^; patient 6 – ALT: r_s_ = -1.00, p = 0.0028, AST: r_s_ = -1.00, p = 0.0028). Corticosteroid doses were also tapered following start of ECP (**Figures 4A, B**). At the individual level, most patients achieved a ≤ 75% corticosteroid dose reduction compared to baseline dosage (at start of ECP) by week 4-6 and near-discontinuation by week 8-9 (**Figure 4A**). Aggregation on cohort level showed that a median corticosteroid dose reduction to 25% (IQR: 20.7 - 33.3) of baseline was reached in week 4, and dose reduction to lower than 5% was reached by week 9 (IQR: 1.6 - 4.7) (**Figure 4B**). Aggregation on cohort level (based on the trajectory of the median corticosteroid dose reduction) confirmed the observed patient level dose reductions.

## Discussion

This retrospective monocentric study demonstrates that ECP can be used as a supportive element and potential salvage therapy to induce clinical remission in severe ICI-induced colitis and hepatitis in patients, who have failed initial steroid-based therapy. These results support the current findings from recently published trial data and case reports. Recent data from Ertl et al. includes the interim analysis of the largest clinical trial to date (21 patients total, of which 11 did receive ECP). They reported an overall response rate of 93% with most patients achieving a resolution of symptoms (73% resolution, 13% improvement, 7% resolution with sequelae) (19). Further, Ruf et al. reported two steroid-refractory irAE cases that were resolved after ECP initiation (22).

In our study, patients with steroid-refractory irColitis showed clinically meaningful improvement within the first two weeks after ECP started and were able to achieve an almost complete remission of symptoms after six weeks. Steroid-refractory hepatitis cases showed a steady decline of liver enzymes over 4-6 weeks after ECP initiation, suggesting that ECP’s therapeutic effects may take several weeks to achieve their full potential. Interestingly, the kinetics of transaminases could provide mechanistic insight: their biological half-lives (AST 16-18h, ALT 42-48h) are considerably shorter than the observed 4-6-week decline, suggesting progressive reduction in ongoing hepatocyte injury rather than immediate cessation of inflammation. This pattern also supports ECP’s mechanism of gradual immune modulation. To assess ECP as potential salvage therapies, it is important to contextualize ECP among other therapeutic options. For steroid-refractory irColitis, tumor necrosis factor-α (TNF-α) antagonists like infliximab and gut-selective vedolizumab have shown high efficacy, achieving clinical response or remission in most patients (24–26). However, it is important to note that TNF-α inhibitors typically require 2 weeks to induce symptom reduction and 7-8 weeks for complete normalization (25). Given that our patients received ECP as an addition to ongoing immunosuppressive therapy, the temporal overlap between ECP initiation and the expected delayed response window of TNF-α inhibitors complicates attribution of clinical improvement to a single modality. The temporal association between ECP initiation and clinical improvement across all patients, combined with the rapidity of response in several cases, suggests that ECP may provide additive or synergistic therapeutic benefit in this refractory population. This is also the case for patients with irHepatitis, in which liver enzymes had already shown some transient decline prior to ECP after the addition of tacrolimus (Patient 5) and MMF (Patient 6). Nevertheless, this decline was not sustained, and transaminase levels remained in a markedly pathological range, indicating that the existing immunosuppressive regimen was insufficient to achieve adequate biochemical control. The subsequent, steady decline of liver enzymes after ECP initiation, which enabled the rapid tapering of concomitant immunosuppressants, supports the role of ECP in achieving stable disease control. This observation is particularly relevant when considering that for most patients with severe irHepatitis, the addition of MMF leads to normalization of elevated liver enzymes (AST/ALT levels to grade ≤1) in the majority of cases (27–29). These options are recommended in guidelines and were utilized in our patients prior to ECP. However, some patients either do not respond, cannot tolerate side effects, or cannot be weaned off corticosteroids due to recurring inflammation. For this subset of patients ECP offers a unique immunomodulatory approach rather than broad immunosuppression. In contrast to classical immunosuppressants, ECP has a favorable safety profile with low infection risk and no known organ toxicities. Moreover, an important advantage is preservation of anti-tumor immunity. Recent preclinical work by Braun et al. demonstrated that, other than high-dose corticosteroids and/or anti TNF-α which impaired anti-PD-1 cancer control, ECP controlled immune toxicity without compromising tumor-specific T cell responses or ICI efficacy (17). However, larger studies are required to elucidate remaining uncertainty on the effect of ECP on immunotherapy outcomes. Consistently, none of our patients showed rapid cancer progression during or after ECP treatment. Although our sample size is comparably small, this observation is encouraging and supports the current view that ECP does not impair melanoma outcomes.

## Limitations

This retrospective monocentric study has limitations given its design. With only six patients, it is not possible to generalize the efficacy rate of ECP or to definitively distinguish its effects from accompanying treatments. However, each patient served as his/her own control in the sense that their colitis or hepatitis was inadequately responsive to standard therapies, and a sustained improvement was only observed after ECP was introduced, suggesting a temporal association. Another limitation is that we did not perform endoscopic or histological assessments after ECP (for colitis cases) or repeated biopsies for hepatitis, so our evaluation of response relies on clinical and laboratory surrogates. Nonetheless, the use of objective criteria (CTCAE grading) strengthens our outcome assessment.

## Conclusion and perspective

Despite the small sample size, our findings add to a growing body of evidence that ECP may be a viable option for steroid-refractory or -intolerant irAEs. At present, there is little comparative research on second-line treatments for these conditions. To address this gap, a prospective side effect registry immune-oncology “SERIO” has been designed to compare ECP with other immunosuppressive therapies for steroid-refractory irAEs (30). Such registries and resulting studies will be critical to determine optimal positioning of ECP and other salvage therapies in current treatment algorithms. Key questions include the ideal timing of ECP initiation (early vs. late second line), the optimal treatment schedule and duration, and long-term outcomes including relapse rates of irAEs and any impacts on cancer survival.

In conclusion, ECP appears to be an effective salvage therapy for irColitis and irHepatitis, capable of inducing clinical remission with a favorable safety profile. Across six patients, ECP was associated with prompt improvement in gastrointestinal symptoms and liver enzymes, corticosteroid tapering, and sustained disease control over more than one year. This retrospective monocenter study, together with limited literature reports, suggests that ECP modulates the immune response to resolve inflammation while preserving anti-tumor immunity. Given the severity of steroid-refractory irAEs and the lack of alternative options in some situations, ECP should be considered at specialized centers, and referral for ECP can be made early during refractory irColitis or irHepatitis. Larger prospective studies and case series are warranted to better define response rates, predictive factors, and to formally establish ECP’s role in the management of irAE in melanoma patients.

## Data Availability

The datasets presented in this article are not readily available because privacy and protection of sensitive data. De-identified data may be made available from the corresponding author upon reasonable request and in line with institutional and data protection regulations.
